# Early Diffuse Alveolar Hemorrhage Revealing Leptospirosis Without Multiorgan Failure: A Case Report and Literature Review

**DOI:** 10.7759/cureus.115783

**Published:** 2026-09-04

**Authors:** Vincent Paternostre, Marc Valette, Frederic Martino, Laurent Camous

**Affiliations:** 1 Intensive Care Unit, Université Catholique de Louvain, Brussels, BEL; 2 Intensive Care Unit, Centre Hospitalier Universitaire de la Guadeloupe, Les Abymes, FRA

**Keywords:** infectious disease, medical intensive care unit (micu), neglected (re)emergent tropical disease, pneumology, tropical disease

## Abstract

Diffuse alveolar hemorrhage is a severe manifestation of leptospirosis, typically occurring in advanced stages of the disease and often associated with multiorgan failure. We report the case of a 44-year-old man without significant medical history admitted for acute febrile illness, hypotension, and respiratory symptoms occurring six days after initial symptom onset. Laboratory findings showed thrombocytopenia, elevated inflammatory markers, hepatic cytolysis, and mild acute kidney injury. Chest imaging demonstrated bilateral ground-glass opacities. Bronchoalveolar lavage (BAL) confirmed alveolar hemorrhage, revealing 1,300,000 red blood cells/mm³ with a neutrophil-predominant profile and negative microbiological investigations. Polymerase chain reaction testing confirmed leptospirosis. The patient was treated with intravenous ceftriaxone followed by oral amoxicillin, resulting in rapid clinical and biological improvement.

This case highlights an atypical pulmonary presentation of leptospirosis without extra-respiratory involvement. Leptospirosis lung involvement should probably be considered in patients presenting with febrile illness, pulmonary infiltrates, and thrombocytopenia, in endemic settings, even without jaundice or severe renal impairment.

## Introduction

Leptospirosis is a widespread zoonotic infection caused by spirochetes of the genus *Leptospira*, transmitted through exposure to water or soil contaminated with the urine of infected animals [1]. The disease represents a significant global health concern, with an estimated one million severe cases annually [2]. Clinical presentation ranges from a mild self-limited febrile illness to severe multiorgan failure. While hepatic and renal involvement have historically been considered the hallmarks of severe disease (Weil disease), pulmonary manifestations are increasingly recognized as major determinants of morbidity and mortality [1,3-5].

Pulmonary involvement is pleomorphic, from mild respiratory symptoms to acute respiratory distress syndrome (ARDS) and diffuse alveolar hemorrhage. Diffuse alveolar hemorrhage was historically described as a separate entity associated with high mortality rates, exceeding 50% in some series [5,6]. Importantly, pulmonary manifestations could occur independently of severe hepatic or renal dysfunction, suggesting distinct underlying mechanisms [4,5].

## Case presentation

A 44-year-old man without a significant medical past was admitted for febrile respiratory failure. Symptoms had begun six days prior with fever, diffuse myalgia, productive cough, and general condition impairment. On the day of hospital admission, he reported blood-tinged sputum. Anamnesis showed environmental exposure through fishing, gardening, and walking in mangrove areas.

On admission, the patient was febrile (39°C), with blood pressure of 80/50 mmHg, heart rate of 110 beats per minute, respiratory rate of 29 breaths per minute, and oxygen saturation of 94% on 3 L/min of oxygen via nasal cannula. Initial fluid resuscitation resulted in partial hemodynamic improvement. Physical examination revealed no neurological deficit and no signs of cardiac failure. Laboratory investigations at ICU admission are reported in Table 1.

**Table 1:** Biological parameters of the patient at ICU admission. Normal values are displayed in the last column. BAL: bronchoalveolar lavage

Parameter	Result	Reference range
Hemoglobin	8.6 g/dL	13.0–17.0 g/dL
Platelet count	56 ×10⁹/L	150–400 ×10⁹/L
C-reactive protein (CRP)	>200 mg/L	<5 mg/L
Aspartate aminotransferase ( AST)	298 U/L	<40 U/L
Alanine aminotransferase ( ALT)	110 U/L	<45 U/L
Total bilirubin	15 µmol/L	3–21 µmol/L
Lactate dehydrogenase (LDH)	555 U/L	120–250 U/L
Creatinine	82 µmol/L	60–110 µmol/L
Urea	5.5 mmol/L	2.5–7.5 mmol/L
Lactate	0.9 mmol/L	0.5–2.0 mmol/L
Arterial pH	7.48	7.35–7.45
Prothrombin time (PT)	90%	70–100%
Activated partial thromboplastin time ratio (aPTT ratio)	1.02	0.8–1.2
Fibrinogen	6.86 g/L	2.0–4.0 g/L
BAL red blood cells	1,300,000 cells/mm³	Absent or minimal
BAL leukocytes	2,000 cells/mm³	Variable
BAL differential count	Marked neutrophilic predominance	Predominantly macrophages

Of note, the patient had mild acute kidney injury consistent with functional renal failure. Chest imaging showed bilateral pulmonary infiltrates, and computed tomography revealed multifocal ground-glass opacities (Figure 1). The association of hemoptysis and anemia raised the suspicion of diffuse alveolar hemorrhage.

**Figure 1:**
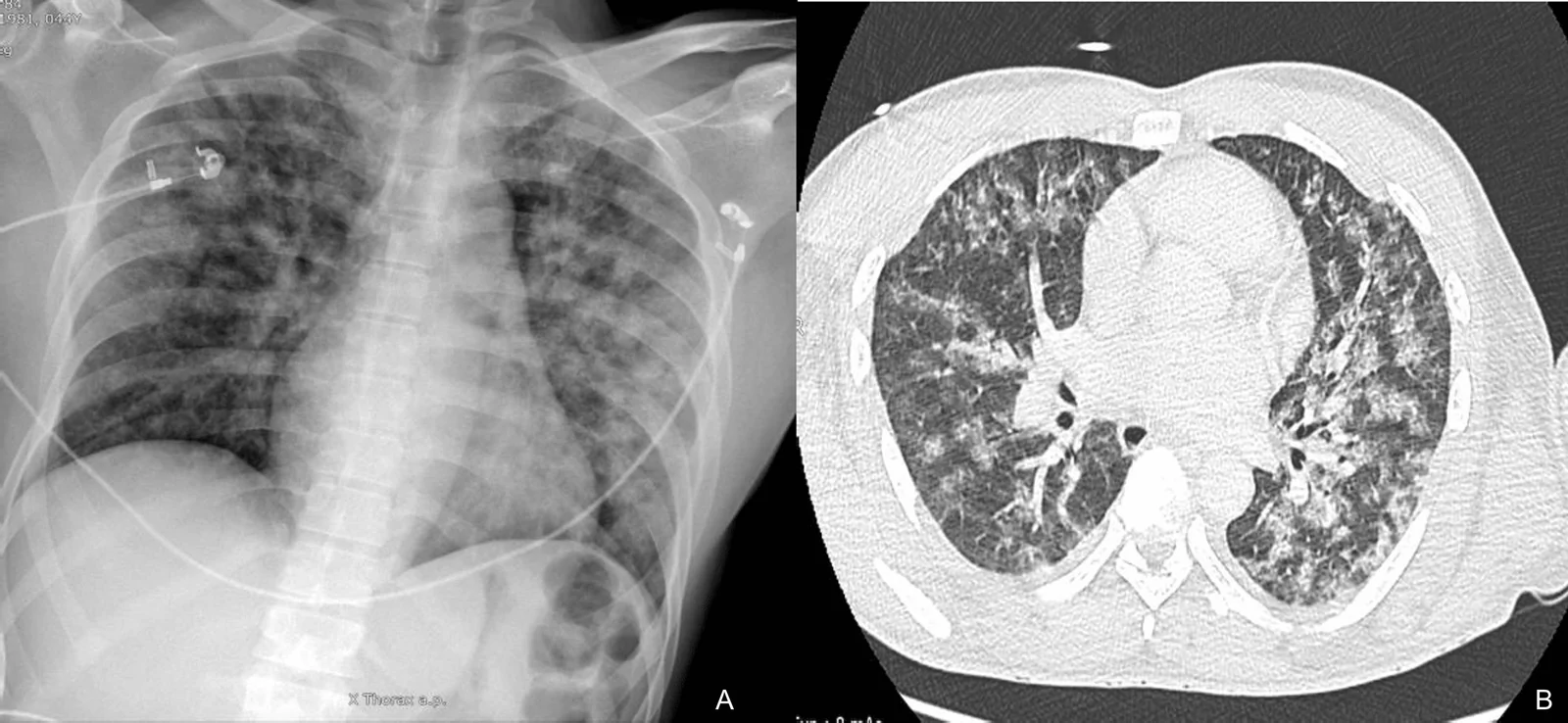
Chest X-ray and CT slices of the patient. Panel A shows bilateral pulmonary infiltrates; Panel B shows a thoracic CT with multifocal ground-glass opacities.

Bronchoscopy with bronchoalveolar lavage (BAL) confirmed alveolar hemorrhage, with cytological analysis reporting 1,300,000 red blood cells/mm³ and 2,000 leukocytes/mm³ with marked neutrophilic predominance.

Microbiological diagnostic investigations (BAL culture, viral and bacterial film array with polymerase chain reaction (PCR)) were negative, supporting a hemorrhagic process rather than infection.

Blood PCR testing for *Leptospira* was positive. All other microbiological investigations were negative. Empirical antibiotic therapy was initiated with a third-generation cephalosporin combined with spiramycin to cover atypical pathogens in the context of an initial presentation consistent with severe community-acquired pneumonia. Once leptospirosis was confirmed by PCR, treatment was streamlined to intravenous ceftriaxone followed by oral amoxicillin for a total of seven days.

The patient showed rapid clinical improvement. Hemodynamic status stabilized, respiratory symptoms improved, and laboratory parameters normalized. No recurrence of hemoptysis occurred, and the patient was discharged.

## Discussion

Leptospirosis-associated pulmonary involvement is characterized by severe endothelial dysfunction and hemorrhagic manifestations. The primary pathophysiological mechanism involves diffuse injury to the vascular endothelium, leading to increased permeability and disruption of the alveolar-capillary barrier [4]. This endothelial damage is part of a broader systemic process, as leptospirosis is known to induce widespread vascular injury affecting multiple organs; histopathological studies have demonstrated such systemic involvement, including cardiac tissue [7]. In the lungs, histopathological studies of deceased patients have reported intra-alveolar hemorrhage with minimal inflammatory infiltrates, supporting the hypothesis of immune-mediated vascular injury rather than direct bacterial invasion [4,5].

Leptospiral components may trigger an exaggerated host immune response. Elevated levels of pro-inflammatory cytokines, including tumor necrosis factor alpha and interleukin-6, have been associated with disease severity and contribute to endothelial damage and capillary leakage [2]. Additionally, immune complex deposition and complement activation may further amplify vascular injury [5]. These mechanisms could be involved in pulmonary damage observed in some patients, even in the absence of high bacterial burden.

Thrombocytopenia, frequently observed in leptospirosis, plays a key role in the development of hemorrhagic complications. Platelet dysfunction, combined with endothelial injury, creates a pro-hemorrhagic state amplifying diffuse alveolar hemorrhage. Although coagulation abnormalities are typically less pronounced than in other hemorrhagic conditions, they may also contribute to disease severity [5]. Emerging data suggest that platelet-endothelium interactions and microvascular dysregulation play a critical role in amplifying vascular permeability and bleeding risk in inflammatory conditions, potentially contributing to the severity of pulmonary hemorrhage in leptospirosis [8].

In our case, BAL was the key diagnostic tool, establishing the diagnosis by cytological analysis. The neutrophil-predominant cellular profile, in the absence of an identifiable lung pathogen, is consistent with an acute inflammatory and hemorrhagic process. These findings reinforce the concept that leptospiral pulmonary involvement is primarily driven by vascular injury and immune dysregulation rather than direct microbial proliferation within the alveolar space.

Clinically, leptospiral pulmonary hemorrhage syndrome has been described by the association of acute dyspnea, hemoptysis, and rapidly progressive hypoxemia. Pathological radiological findings usually show bilateral ground-glass opacities and alveolar infiltrates [5,6]. The state-of-the-art review on leptospirosis lung involvement by Marchiori et al. emphasizes that high-resolution computed tomography mostly showed ground-glass attenuation, consolidations, nodular opacities, and even “crazy-paving” patterns [5]. These data reflect that lung damage is mostly due to intra-alveolar hemorrhage rather than inflammatory mechanisms.

Importantly, pathological radiologic abnormalities could appear early in the disease course, even within the first days of hospitalization, and can even precede other severe symptoms [5].

In our case, diffuse ground-glass opacities were observed without severe clinical manifestations. Furthermore, pulmonary hemorrhage may occur without marked radiographic abnormalities or could mimic diseases (viral pneumonia or ARDS), making diagnosis challenging [5]. This highlights the importance of early imaging and of considering leptospiral pulmonary involvement in patients presenting with atypical febrile pneumonia in endemic areas.

The large multicentric retrospective study by Allyn et al. provides important insights into the clinical heterogeneity of severe leptospirosis [9]. By analyzing ICU patients from both tropical and temperate regions in France, the authors identified three distinct clinical phenotypes: a hepato-renal form corresponding to classical Weil disease, a moderately severe form with limited organ dysfunction, and a very severe phenotype characterized by neurological, respiratory, and cardiovascular failure with high mortality (30%).

Notably, pulmonary involvement, including hemoptysis, was present in all subgroups and was not independently associated with severe hepatic or renal dysfunction. One striking feature of our case was the absence of liver failure and the mild renal involvement. This observation further supports the concept that pulmonary involvement is probably underdiagnosed and is not a simple marker of global severity.

The study by Fonseka et al., conducted in an endemic setting in Sri Lanka, reported pulmonary hemorrhage in up to 38% of severe cases and was a major predictor of mortality, particularly when associated with hemodynamic instability [10]. A significant acute reduction in hemoglobin, reflecting pulmonary hemorrhage, was observed in nearly 80% of patients with pulmonary involvement and was associated with poor outcomes. Hospital mortality reached over 35% in patients with pulmonary hemorrhage and increased further when combined with fulminant hepatic failure.

Importantly, in the study by Fonseka et al., pulmonary hemorrhage almost invariably occurred in the context of severe systemic disease with multiple organ dysfunction, frequently requiring intensive care, mechanical ventilation, and advanced supportive therapies such as extracorporeal membrane oxygenation (ECMO) [10].

In contrast, our report showed severe diffuse alveolar hemorrhage occurring in the absence of severe organ failures or hemodynamic collapse. This discrepancy further supports the notion that pulmonary involvement in leptospirosis is frequent, underdiagnosed, and not a late evolution of the disease.

The retrospective series of Paganin et al. reported that pulmonary involvement was also generally associated with severe disease, acute respiratory failure, and high mortality rates (40%) [11]. Dyspnea and oliguria were identified as predictors of severity, and most patients with pulmonary manifestations required aggressive supportive care.

Further supporting the central role of respiratory involvement, Vandroux et al. analyzed a cohort of ICU patients with leptospirosis and found that 23% developed moderate to severe ARDS, secondary to intra-alveolar hemorrhage in most of the cases, with a mortality rate of 23% [12]. Most patients required invasive mechanical ventilation, and a subset required ECMO for refractory hypoxemia. These findings reinforce the concept that pulmonary involvement in leptospirosis is typically severe, frequently progresses to ARDS, and is often associated with multiorgan failure.

Interestingly, despite the severity of presentation, the prognosis of leptospirosis-related ARDS was better than that of ARDS from other causes, suggesting a distinct and potentially reversible pathophysiology [12]. However, in this cohort, pulmonary involvement was almost invariably associated with significant systemic severity, including high Sequential Organ Failure Assessment (SOFA) score and Simplified Acute Physiology Score II (SAPS II), renal failure, and hemodynamic instability. This highlights a strikingly atypical trajectory compared to the patterns described by Vandroux et al., further supporting the hypothesis that alveolar hemorrhage in leptospirosis may, in some cases, precede or even occur independently [12].

Most reported cases described diffuse alveolar hemorrhage occurring during advanced stages of the disease and often associated with multiorgan failure. For example, Gouveia et al. reported high mortality rates associated with pulmonary hemorrhage [6], while Barnacle et al. described a case requiring ECMO due to severe respiratory failure [13].

In contrast, our patient with confirmed diffuse alveolar hemorrhage did not worsen with the need for mechanical ventilation or ARDS and quickly clinically improved. A possible explanation is the early initiation of antimicrobial therapy, which has been shown to improve clinical outcomes and shorten disease duration in leptospirosis [14].

A limited number of reports have described early pulmonary manifestations of leptospirosis. Although data remain scarce, these reports suggest that pulmonary hemorrhage may occasionally be the initial presentation of the disease. Our case further strengthens this evidence and suggests that diffuse alveolar hemorrhage may occur earlier in the disease course than traditionally described, preceding significant extrapulmonary organ dysfunction.

The favorable outcome observed in this case may be explained by early diagnosis and prompt initiation of antibiotic therapy. Molecular diagnostic techniques such as PCR allow early detection of infection and timely treatment. Early administration of antibiotics, particularly beta-lactams such as ceftriaxone, has been associated with improved outcomes [1]. In addition, close monitoring and supportive care likely contributed to preventing progression toward severe respiratory or multiorgan failure.

The potential role of immunomodulatory therapies in leptospirosis-associated pulmonary hemorrhage remains debated. Given the immune-mediated nature of endothelial injury, corticosteroids have been proposed as adjunctive therapy, particularly in severe diffuse alveolar hemorrhage. Observational data, including a case series [15], suggested a significant reduction in mortality with bolus methylprednisolone in pulmonary leptospirosis. However, the only randomized controlled trial evaluating high-dose corticosteroids in severe leptospirosis, including patients with pulmonary involvement, failed to demonstrate a survival benefit and was associated with an increased risk of nosocomial infections [16]. A recent systematic review and meta-analysis confirmed that evidence remains insufficient to support routine corticosteroid use in this setting [17]. In our case, no immunomodulatory therapy was administered, and the favorable outcome was achieved with antibiotic therapy alone, suggesting that in early-stage disease without refractory organ failure, targeted antimicrobial treatment may be sufficient. Prospective studies designed to evaluate immunomodulatory strategies in leptospiral diffuse alveolar hemorrhage are warranted.

This case emphasizes the importance of considering leptospirosis in patients presenting with febrile illness, thrombocytopenia, and pulmonary infiltrates, even in the absence of classic hepatic or renal involvement. Early recognition of diffuse alveolar hemorrhage is essential, as delayed diagnosis may lead to rapid clinical deterioration. Furthermore, awareness of atypical presentations may help clinicians initiate appropriate diagnostic and therapeutic strategies at an earlier stage of the disease.

## Conclusions

Diffuse alveolar hemorrhage is a life-threatening pulmonary manifestation of leptospirosis that may be underrecognized, particularly when it occurs in the absence of classical hepatorenal dysfunction. This case illustrates an atypical early pulmonary presentation occurring in the absence of severe respiratory failure or multiorgan dysfunction, with a favorable clinical outcome following prompt treatment.

This observation supports the concept that pulmonary involvement in leptospirosis may represent an early and partially independent disease manifestation rather than a late complication of systemic severity. Increased awareness of such atypical presentations is essential, as early recognition and timely management may help prevent progression to severe disease and improve patient outcomes.
